# Inhibitory Control in Speech Comprehension among Dai–Han Bilingual Children

**DOI:** 10.3389/fpsyg.2017.01391

**Published:** 2017-08-17

**Authors:** Yun Tao, Zhi Liu, Tobias Tempel, Rui Chen, Xie Ma, Xiaoxi Wang, Yan Liu, Yongxia Qu

**Affiliations:** ^1^School of Educational Science and Management, Yunnan Normal University Kunming, China; ^2^Key Laboratory of Educational Informatization for Nationalities, Ministry of Education, Yunnan Normal University Kunming, China; ^3^Fachbereich I – Psychologie, University of Trier Trier, Germany; ^4^College of Preschool Education and Special Education, Kunming University Kunming, China; ^5^Faculty of Foreign Languages and Cultures, Kunming University of Science and Technology Kunming, China

**Keywords:** Dai–Han bilinguals, proficient, primary school students, response inhibition, attentional control

## Abstract

We aimed to investigate differences in inhibitory control ability between proficient and non-proficient Dai–Han bilinguals. Two experiments used a combined *stimulus–stimulus and stimulus–response compatibility paradigm* for this purpose. Participants were Dai–Han bilingual primary-school students selected from a Dai-speaking town in Yunnan province, China. In Dai language interference condition, participants were asked to complete a picture category task. Results showed that the effect of attentional control for non-proficient bilinguals (NPBs) was significantly greater than that for proficient bilinguals (PBs), while the effect of response inhibition was not. This implied that a difference in inhibitory control between PBs and NPBs appeared at the attention control stage when interference by the Dai lexicon emerged. In Han language interference condition, however, participants were also asked to complete the same task. Results showed that the effect of response inhibition for NPBs was significantly greater than that for PBs, but the effect of attentional control was not. This demonstrated that a difference in inhibitory control emerged at the response inhibition stage when interference by the Han lexicon emerged. This pattern of results is opposite to previous researches, which indicated that the difference between PBs and NPBs occurred at the response inhibition stage under first language condition, whereas at the attentional control stage under second language (L2) condition. Based on these, this study suggests that Dai–Han bilinguals showed a remarkable L2 advantage. In addition, results showed that response times (RTs) of PBs were faster than RTs of NPBs while confounding variables (e.g., intelligence, etc.) were under control. This indicates that the inhibitory control ability of the PBs is superior to that of NPBs in this study.

## Introduction

Lexicon acquisition is one of the most important aspects of elementary school learning and a fundamental basis of speech comprehension. Speech organization is an intermediary process wherein target concepts are transferred into articulated expressions. This process requires proper lexicon selection. To better understand the basic processes of speech comprehension, many researchers in the philological and psychological fields have examined lexicon selection processes specifically in bilinguals, as bilinguals are believed to possess two independent mental lexicons (Weinreich, 1953; Heuven et al., 1998; Costa et al., 1999; Evans et al., 2002).

In linguistics, bilinguals are defined as individuals able to use two standard languages (Grosjean, 1982). Normally, languages are acquired in succession. The language a person acquires first is considered their *first language* (L1) and the language acquired second is called the *second language* (L2). A bilingual is one who can speak two languages. Based on their mastery of L2, bilinguals can be further categorized as either proficient bilingual (PB) or non-proficient bilingual (NPB). To study the selection processes of mental lexicons among bilinguals, cognitive researchers have typically used language-related tasks where participants are requested to select required words from one of their mental lexicons for representation. For instance, a study by Li et al. (2006) demonstrated differences in the lexicon selection mechanism of PBs and NPBs. Whereas PBs directly access the required mental lexicon, NPBs tend to rely heavily on L1 as a medium to obtain access to the mental lexicon of L2.

The need to initially access L1 for NPBs has been demonstrated using language-switching paradigms. Specifically, several studies have found an asymmetry of switch costs for NPBs – namely, switch costs are higher when they switch from L2 to L1 as compared to switching from L1 to L2. In contrast, switch costs for PBs have been found to be symmetrical (Meuter and Allport, 1999; Costa and Santesteban, 2004; Costa et al., 2006). Burgess and Shallice (1996) suggest that inhibitory control accounts for that asymmetry in switch costs. Bilinguals can readily accomplish lexicon selection (i.e., retrieve the target language) by inhibiting retrieval of the non-target language. This might indicate that activation of the target language is greater than activation of the non-target language. For NPBs, activation of their L1 initially is higher. Therefore, inhibition requires stronger effort, resulting in an asymmetry in switch costs. In contrast, activation of both L1 and L2 is equally high in PBs when they target either language, resulting in symmetrical switch cost (e.g., Li et al., 2006).

The Inhibition Control Model by Green (1998) explains why switch costs are symmetrical in PBs and asymmetrical in NPBs. This model assumes that both languages are activated in the process of lexicon selection for bilinguals, which results in interference. To resolve this competition, three elements need to be in operation: the concept, the semantic system, and the verbal task schema. Specifically, input information is transferred from a conceptual representation into the mental lexicon – which represents a broader bilingual lexicon-semantic system – through the concept. The language task schema controls the output of the bilingual semantic system through competition. The supervisory attention system activates a relevant (i.e., task-related) language task scheme for processing the input stimulus while inhibiting the unrelated language task scheme. Simultaneously, the activated language task scheme selects the task-related lexicon and inhibits any unrelated lexicons. This model emphasizes two points: First, the higher the initial activation level, the greater the inhibitory control necessary to prevent processing/output (i.e., to inhibit activation of unrelated lexicons). Second, inhibition persists over a certain delay, depending on the strength of the inhibition – the greater the inhibitory control, the longer it persists. Because PBs are highly proficient in both L1 and L2 the same amount of inhibitory control ability is required to inhibit the task-unrelated language. In contrast, NPBs need less inhibitory control ability to inhibit L2, whereas they need more inhibitory control ability to inhibit L1.

Bilinguals’ inhibitory control ability is reflected in the ease with which they can resolve competition between target and non-target lexicons. Such competition may occur at any time during speech comprehension in a language-switching task, from stimulus input (stimulus presentation) to response output (stimulus category). Competition during input reflects conflicts at the stimulus processing level (i.e., the name of a picture and the word on the picture in this study), which is mainly influenced by language proficiency; whereas competition during output reflects conflicts at the response level (i.e., the position of a picture and the position of the response key in this study), which is mainly influenced by nonverbal inhibitory ability. Bunge et al. (2002) pointed out that conflicts at the input level are related to the directedness of attention. Specifically, when non-task-related language is prompted, attention must be re-directed toward task-related language in order to give the correct response. This stage is called *attentional control*. Conflicts at the output level, on the other hand, are related to the avoidance of automatic responses. In other words, it requires a controlled response instead of a habitual response that is a prompted response must be avoided. This stage is called *response inhibition*. According to Friedman and Miyake (2004), attentional control serves to inhibit attention to non-task-related language, whereas response inhibition serves to inhibit inappropriate prepotent responses. Thus, attentional control resolves conflicts between competitively activated stimulus information, whereas response inhibition comes into play when changing or stopping an inappropriate dominant response is needed. To summarize, the current literature suggests that the asymmetry in switch costs results from inhibitory control, which operates in two stages: attentional control and response inhibition.

The present study examined the inhibitory control of Dai–Han bilingual primary school students. Living mainly in Yunnan, China, Dai possesses both verbal and written languages independent of those of Han. A Zhuang-Dai branch of the Sino-Tibetan languages, the written script of Dai is alphabetic derived from Devanagari (Luo, 2008). Children of Dai communicate in their own language before schooling. Some children may know some Chinese (i.e., Han), which they learn or pick up from radio, TV, and/or movies. When they go to elementary school, they have to learn Han in a formal way, which will help to expand their understanding of the outside world. According to Bloomfield (1933) L1 is the language a person has been exposed to and is able to speak from his birth while L2 refers to the language a person learns to speak or use other than their L1. As far as Dai children are concerned, their L1 is Dai and Han is the L2 they learn to speak when they start schooling. Currently, most Dai regions in China have adopted a bilingual (Dai and Han) teaching approach, the purpose of which is to enable Dai children to gradually use Chinese as their primary language for learning, enhance their communication with other ethnic minorities, and help them better immerge into the Han-culture-oriented social system as well as preserve their Dai language and culture. The sample of 442 students chosen for the experiment lived in a largely homogenous language environment but possessed individual differences in Dai–Han language proficiency as well.

Experiment was conducted with these students to identify when inhibitory control occurs and to determine the difference in inhibitory control between PBs and NPBs at resolving interference of the Dai (L1) or Han (L2) lexicons with current task goals. Cai (2010) found that the Stroop effect in PBs was significantly larger than NPBs while they were asked to name the color in Chinese (L1) but the color-word was presented in English (L2). According to the previously, studies using the Stroop paradigms indicated that inhibitory control occurs at the attentional control stage. This result revealed that the difference of inhibitory control between PBs and NPBs was occurred at the attentional control stage with the L2 as interference. Furthermore, in a study of Han-English PBs and NPBs, Li (2007) found that inhibitory control occurred at the stage of response inhibition with interference by L1, and suggested that the difference of inhibitory control ability is influenced by nonverbal inhibitory ability but not by proficiency of L1. In contrast, inhibitory control occurred at the stage of attentional control with interference by L2. Here, the difference of inhibitory control ability was influenced by proficiency of L2. The PBs’ proficiency level in L2 is assumed to be as high as their proficiency level in L1 in the current study (Dai and Han, respectively). It is therefore expected that PBs require more cognitive resources to inhibit the task-irrelevant language. Nevertheless, inhibiting the task-irrelevant language should be easier for NPBs because they are much less proficient in L2. Laurent and Martinot (2010) also noted that children between the ages of 8 and 10 who have taken a bilingual program at school tend to demonstrate more developed cognitive ability than their monolingual peers. The hypotheses are:

(1) The inhibitory control ability of PBs would be stronger than the inhibitory control ability of NPBs because of the greater bilingual proficiency of PBs.
(2) A difference of inhibitory control between Dai–Han PBs and NPBs would occur in the stage of response inhibition with Dai stimuli (L1).
(3) A difference of inhibitory control between Dai–Han PBs and NPBs would occur in the stage of attentional control with Han stimuli (L2).

Most previous studies have adopted the Stroop(Stroop, 1935), Simon(Simon, 1969; Simon and Berbaum, 1990), or Sustained Attention to Response task (SART) (e.g., Robertson et al., 1997) paradigms to study the stage at which inhibition of lexicon selection occurs. Studies using the Stroop and Simon paradigms indicated that inhibitory control occurs at the attentional control stage. Whereas the Stroop task reflects stimulus–stimulus compatibility (i.e., both color and semantic are involved in one stimulus), the Simon task reflects stimulus–response compatibility (i.e., consistency of position between stimulus and response key). However, studies using SART – which also reflects stimulus–response compatibility – have found that inhibitory control during lexicon selection occurs at the response inhibition stage (e.g., Zhang et al., 1999; Bialystok et al., 2008). Therefore, conclusions about the stage of inhibitory control during lexicon selection might depend on the choice of paradigm used.

To avoid the negative effects of paradigm selection on the results and to examine the differences between the two stages of inhibitory control efficiently, Kornblum (1992) developed a paradigm that is able to examine both stimulus–stimulus and stimulus–response compatibility. This paradigm – called the *dimensional overlap* (DO) *paradigm* – has become the most widely applied paradigm for investigations of a multi-dimensional inhibitory control model. The DO paradigm allows examination of both stimulus–stimulus (i.e., the name of a picture and the word on the picture in this study) and stimulus–response (i.e., the position of a picture and the position of the response key in this study) dimensions. It includes four conditions: ➀ stimulus consistent–response consistent, ➁ stimulus consistent–response inconsistent, ➂ stimulus inconsistent–response consistent, and ➃ stimulus inconsistent–response inconsistent (e.g., Kornblum et al., 1990; Kornblum, 1992, 1994; Kornblum and Lee, 1995). Based on the concept of subtractive method, Kornblum and Lee (1995) propose formulas to calculate the effects of attention control and response inhibition, respectively. More precisely, the formula for the effect of attention control = [(➂-➀) + (➃-➁)]/2 can explain whether a word stimulus presented on a picture interfered with picture naming. The effect of response inhibition = [(➁-➀) + (➃-➂)]/2 can indicate whether the position of presented picture interfered with determining the position of response keys. Liu (2011) used this paradigm and verified the effect of attention control in inhibitory control between Han monolinguals and highly proficient Meng-Han bilinguals. While Liu’s study revealed that the effect of attention control in Han monolinguals was stronger than in highly proficient Meng-Han bilinguals, the focus of the study was on the effect of attentional control stage, rather than on both attention control and response inhibition stages, neither did it examine the stage of inhibitory control during lexicon selection in L1.

## Materials and Methods

In this experiment, participants were asked to classify pictures in two categories. Furthermore, Dai words that were either semantically related or unrelated to the picture were presented with each picture. The response location either matched or did not match the location of the picture on the screen. We assumed that interference of Dai words would affect processing at the response inhibitory stage because Dai was L1. Assuming that PBs possess superior inhibitory ability a greater interference effect on NPBs was also expected.

### Participants

Participants were selected from a sample of 442 Dai–Han bilingual children from a Dai-speaking region in Yunnan province where the majority of the locals speak Dai. All participants volunteered to take part in the experiment. Their average age was 9.39 (*SD* = 0.68). Participants’ performance scores of the Dai language in listening, speaking, reading, and writing fell into a normal distribution. One hundred and twenty children from the highest 27% of the performance distribution were chosen as proficient Dai language participants. They were then tested with the same model of their Han language performance, and 27% of the participants (14 males and 18 females) who scored the highest in the Han language performance were grouped as PB (average age = 9.88, *SD* = 0.79), while the lowest 27% (17 males and 15 females) were assigned to the NPB group (average age = 9.91, *SD* = 0.59). The PB group consisted of 32 children proficient in both Dai and Han. The NPB group consisted of 32 children highly proficient in Dai but with low proficiency in Han. All children were right-handed and had normal or corrected-to-normal vision without color vision deficiencies. Furthermore, to avoid the negative influence of nonverbal IQ on language acquisition, Raven’s Standard Progressive Matrices (SPM) was adopted to measure the difference of nonverbal IQ between PBs and NPBs. There was no significant difference between PBs (average score = 57.28, *SD* = 7.79) and NPBs (average score = 56.11, *SD* = 6.99), *t*(62) = 0.63, *p* = 0.53.

This study was carried out in accordance with the recommendations of Ethical Principles of Psychologists and Code of Conduct from the American Psychological Association with written informed consent for all participants’ guardians. An Ethics Commission at Yunnan Normal University approved the study.

### Material

Two sets of 16 color cartoon pictures (320 × 320 pixels) were prepared: one showing animals, such as dog and cat and the other depicting housework, for example cooking and mopping. In Dai condition, 32 Dai words for both animals (nominal nouns) and housework activities (activity verbs) were provided in purple (RGB: 125, 0, 228; Dai New-1 typeface; size 72).

In Han condition, the 32 words depicting animals and housework tasks were in Han instead of Dai. All words were printed in purple (RGB: 125, 0, 228), using font type of Song, and in size 72. Fifty percent of the pictures were paired with correct Han caption while the other half of the pictures and captions were mismatched.

### Design

The experiment was designed to follow the procedures of a mixed combination of proficiency (PB, NPB), language type (Dai, Han), stimulus consistency (stimulus consistent, stimulus inconsistent), and response consistency (response consistent, response inconsistent), with the latter two factors as repeated measures variables. The dependent variable was response time (RT).

### Procedure

**FIGURE 1:**
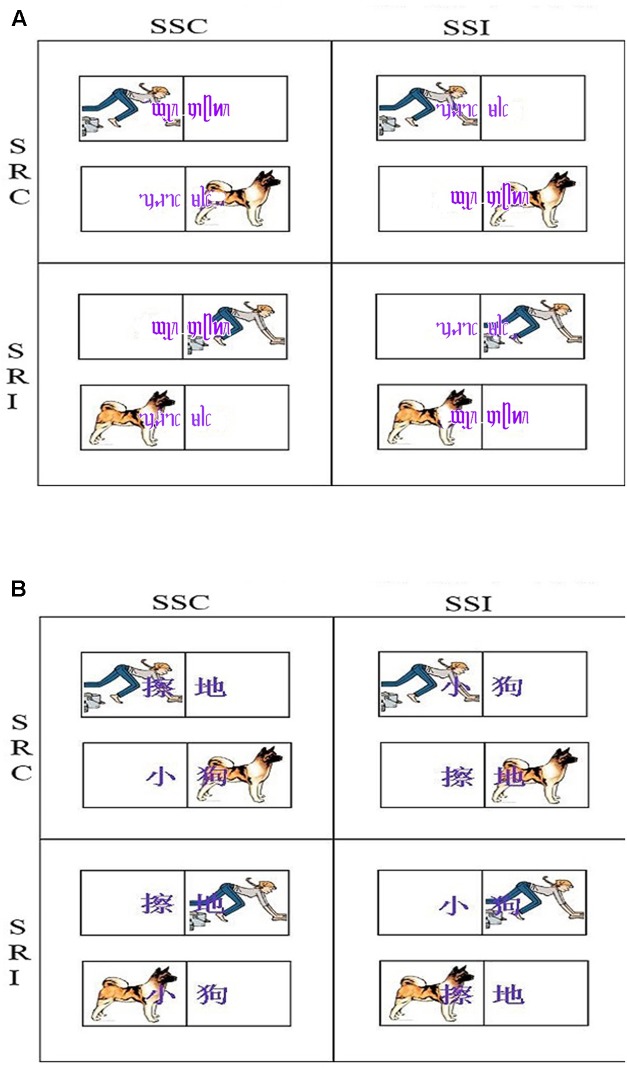
Stimulus–stimulus and stimulus–response compatibility paradigm example. The figure above shows the relational positions of the pictures and key presses. The instructions were as follows: when a nominal picture is shown, press the “L” key; when a verbal picture is shown, press the “A” key. Response keys were counterbalanced, such that another group had nominal pictures assigned to the “A” key, and verbal pictures assigned to the “L.” SSC, stimulus–stimulus consistent; SSI, stimulus–stimulus inconsistent; SRC, stimulus–response consistent; and SRI, stimulus–response inconsistent. P.S., Dai words showed in **(A)**, “
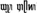
” means mopping and “
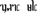
” means puppy. Han words showed in **(B)**, “
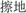
” means mopping and “
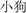
” means puppy.

The DO paradigm was adopted. The stimulus–stimulus dimension was operationalized as the correspondence between lexicons and pictures, and was divided into two levels: consistent and inconsistent. The stimulus–response dimension was operationalized as the correspondence of picture location and key-pressing location, which was also divided into two levels: consistent and inconsistent. Accordingly, there were four conditions: (1) stimulus–stimulus consistent and stimulus–response consistent; (2) stimulus–stimulus consistent and stimulus–response inconsistent; (3) stimulus–stimulus inconsistent and stimulus–response consistent; and (4) stimulus–stimulus inconsistent and stimulus–response inconsistent (**Figures 1A,B**).

E-prime 2.0 developed by Psychology Software Tools was used to process the experiment result. Before the experiment started, the participants were required to sit about two feet away (screen-to-eye).

The instructions for each experimental block were presented on the computer screen. After reading the instructions for the practice phase, participants were requested to press the “Q” key to start the practice. There were four blocks in this experiment. Each block started with 12 practice trials. Once they knew how to do the practice, the participants proceeded to start each experimental phase by pressing the space bar. The pictures used in the practice trials were not the same as those used in the experimental phase. Each trial follows the same procedure: (1) a fixation cross appeared in the center of the screen within a white frame for 1000 ms and (2) a picture popping up on either the left or right side of the screen together with a word appearing in the center of the screen, overlapping the picture. The participants were required to judge whether the picture was of a nominal or verbal category while ignoring its location and the printed word. When a verbal picture was shown, they were asked to press the “A” key with their left index finger, and if a nominal picture was shown, they should press the “L” key with their right index finger. They were also asked to respond as quickly and as accurately as possible. If a participant did not respond within 3000 ms, the computer would automatically show a new trial. The experimental phase consisted of 64 trials with equal numbers of animals and housework pictures randomly presented in each language type. Half of participants began with Dai language interference task, and the rest began with Han. The response key positions were counterbalanced across participants.

## Results

The overall accuracy in this experiment was above 95.28%. Accuracy was not considered as the examined independent variables, we report no further on it in this experiment. All trials with inaccurate responses have been excluded from further analysis. One sample K–S tests showed that all valid RTs followed normal distribution.

A 2 (proficiency) × 2 (stimulus consistency) × 2 (response consistency) ANOVA examined differences in RTs separately in Dai and Han interference task. In Dai condition, we found a significant main effect of proficiency, *F*(1,63) = 609.563, *p* < 0.001, $\eta_{p}^{2}$ = 0.908, SP = 1.000, which indicated that the RTs of PBs were shorter than were those of NPBs. Furthermore, we found a significant main effect of stimulus consistency, *F*(1,63) = 239.742, *p* < 0.001, $\eta_{p}^{2}$ = 0.795, SP = 1.000, which indicated that RTs of stimulus consistent trials were shorter than those of the stimulus inconsistent trials. There was also a significant main effect of response consistency, *F*(1,63) = 19.323, *p* < 0.001, $\eta_{p}^{2}$ = 0.238, SP = 0.991, indicating that RTs for response consistent trials were shorter than those for response inconsistent trials (**Table 1**). The interaction between proficiency and stimulus consistency was also significant, *F*(1,62) = 6.137, *p* = 0.016, $\eta_{p}^{2}$ = 0.090, SP = 0.684. A simple effects analysis indicated that RTs to PBs were shorter than RTs to NPBs in both stimulus consistent and stimulus inconsistent trials (*p*s < 0.001). Response times for the stimulus consistent trials were shorter than the RTs for the stimulus inconsistent trials for both PBs (*p* < 0.001) and NPBs (*p* < 0.001). In Han condition, the main effect of proficiency was significant, *F*(1,63) = 366.156, *p* < 0.001, $\eta_{p}^{2}$ = 0.855, SP = 1.000, which indicated that RTs of PBs were shorter than RTs of NPBs. The main effect of response consistency was significant, *F*(1,63) = 73.998, *p* < 0.001, $\eta_{p}^{2}$ = 0.544, SP = 1.000, which indicated that RTs for response consistent trials were shorter than RTs for response inconsistent trials. The main effect of stimulus consistency was also significant, *F*(1,63) = 72.242, *p* < 0.001, $\eta_{p}^{2}$ = 0.538, SP = 1.000, which indicated that RTs for stimulus consistent trials were shorter than RTs for stimulus inconsistent trials (also see **Table 1**). The interaction between proficiency and response consistency was significant, *F*(1,62) = 24.886, *p* < 0.001, $\eta_{p}^{2}$ = 0.286, SP = 0.998. A simple effect analysis revealed that RTs of PBs were shorter than RTs of NPBs in both response consistent and response inconsistent trials (*p*s < 0.001). RTs for response consistent trials were shorter than those for response inconsistent trials for both PBs (*p* = 0.013) and NPBs (*p* < 0.001). Specifically, the difference between response consistent and response inconsistent trials was larger for NPBs than for PBs (*p* < 0.001). Finally, the interaction of stimulus consistency and response consistency was significant, *F*(1,62) = 4.534, *p* = 0.037, $\eta_{p}^{2}$ = 0.068, SP = 0.554. A simple effect analysis showed that the difference between stimulus consistent and stimulus inconsistent trials was larger for response inconsistent trials (*p* < 0.001) than for response consistent trials, although it was still significant for response consistent trials (*p* < 0.001). The remaining interactions were not significant (*F*s < 3.894, *p*s > 0.053).

**Table 1:** Means and standard deviations of participants, RTs in different experimental conditions (ms).

Language type	Proficiency	*n*	Stimulus consistent		Stimulus inconsistent	
				
			Response consistent	Response inconsistent	Response consistent	Response inconsistent
Dai	PB	32	681 (69)	703 (79)	802 (73)	817 (75)
	NPB	32	1080 (82)	1112 (79)	1239 (103)	1277 (108)
Han	PB	32	693 (100)	721 (102)	760 (82)	790 (95)
	NPB	32	1067 (85)	1152 (126)	1117 (84)	1245 (105)


*PBs, proficient Dai–Han bilingual primary school children and NPBs, non-proficient Dai–Han bilingual primary school children.*

To ensure that the difference of inhibitory control ability between PBs and NPBs was caused by superior inhibitory control ability of PBs, a baseline was assigned to compare the RT of the two groups. In Dai language interference condition, the experimental condition of the current trial, which was the same as the last trial (i.e., both of the last and current trials were in the stimulus-consistent + response consistent condition), was used as a baseline. Furthermore, the experimental condition of the current trial, which was different from the last trial (i.e., the last one was in the stimulus-consistent + response consistent condition, while the current one was in the stimulus-inconsistent + response consistent condition), was considered a non-baseline. A comparison of the differences in baseline RT and inhibitory control cost (i.e., non-baseline RT subtracted by baseline RT) of the PBs and NPBs showed that the difference between PBs (728 ms) and NPBs (764 ms) in their baseline RT was not significant [*t*(62) = 1.73, *p* = 0.09] whereas the difference between PBs (66 ms) and NPBs (834 ms) in their inhibitory control cost was significant [*t*(62) = 38.66, *p* < 0.01]. In Han language interference condition, moreover, the difference between PBs (699 ms) and NPBs (747 ms) in their baseline RT was not significant (*t* = 1.83, *p* = 0.07) while the difference between PBs (97 ms) and NPBs (7944 ms) in their inhibitory control cost was significant (*t* = 28.32, *p* < 0.01). This result confirms that the advantage of PBs’ inhibitory control ability is the main cause of the significant difference of RT between PBs and NPBs in all experimental conditions (i.e., the existence of differences between PBs and NPBs in their inhibitory control ability).

**FIGURE 2:**
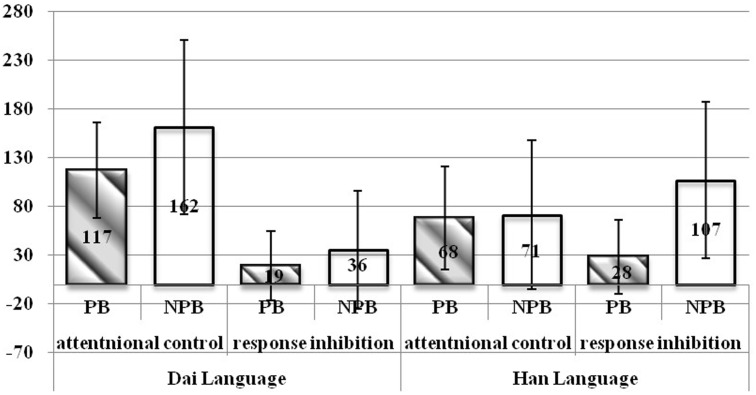
Mean effect of different inhibitory control stages. Effect of attention control = RTSI–RTSC and effect of response inhibition = RTRI–RTRC (Kornblum and Lee, 1995). SI, stimulus inconsistent; SC, stimulus consistent; RI, response inconsistent; and RC, response consistent.

In addition, the effects of inhibitory control in both attentional control and response inhibition stages were calculated according to Kornblum and Lee’s (1995) formula. A 2 (proficiency) × 2 (language type) × 2 (stages of inhibitory control) ANOVA examined differences in effect. We found a significant main effect of proficiency, *F*(1,62) = 19.844, *p* < 0.001, $\eta_{p}^{2}$ = 0.242, SP = 0.992, which indicated that the effect of NPBs was greater than those of PBs. Furthermore, we found a significant main effect of language type, *F*(1,62) = 6.811, *p* = 0.011, $\eta_{p}^{2}$ = 0.099, SP = 0.729, which indicated that the effect of Dai language was greater than those of Han language. There was also a significant main effect of inhibitory control stages, *F*(1,62) = 50.382, *p* < 0.001, $\eta_{p}^{2}$ = 0.448, SP = 1.000, indicating that the effect in the stage of attentional control was greater than those in the stage of response inhibitory. The interaction between language type and inhibitory control stages was significant, *F*(1,62) = 34.259, *p* < 0.001 $\eta_{p}^{2}$ = 0.356, SP = 1.000. Furthermore, an interaction between proficiency, language type, and inhibitory control stages was also significant, *F*(1,62) = 7.520, *p* = 0.008 $\eta_{p}^{2}$ = 0.108, SP = 0.770 (**Figure 2**). A simple effects analysis indicated that in Dai condition, the effect of attentional control for NPBs was significantly greater than that for PBs, while the effect of response inhibition was not. In Han condition, however, the effect of response inhibition for NPBs was significantly greater than that for PBs, but the effect of attentional control was not. Furthermore, for both PBs and NPBs, the effect of attentional control in Dai language was significantly greater than in Han language. The effect of response inhibitory in Dai language, however, was significantly smaller than in Han language only for NPBs. The rest of the interactions were not significant (*F*s < 2.914, *p*s > 0.093).

## Discussion

According to all above results, the inhibitory control of PBs was greater than that of NPBs, which supports the first hypothesis of the study. However, the second and third hypotheses were opposite to results. In regard to the second hypothesis, specifically, only the effect of attentional control was smaller for PBs than for NPBs in Dai condition. Thus, a difference of inhibitory control emerged with regard to attentional control. In addition, the greater effect of stimulus consistency compared to response consistency indicates that interference by Dai stimuli on performance in the DO paradigm was greater than interference by response location. It would be appropriate to assume that more cognitive resources were occupied at the attentional control stage for NPBs than for PBs. The smaller effect of response consistency may have precluded a moderating effect of proficiency on the response inhibition effect to emerge. This finding matches the previous study using L2 stimuli by Martin-Rhee and Bialystok (2008) who did find differences in inhibition control between PBs and NPBs to occur mainly at the attentional control stage. Similarly, a study by Liu (2011) found that inhibitory control of Meng-Han bilinguals was stronger as compared to Han monolinguals at the attentional control stage when using Han (i.e., L2) stimuli (see also Li, 2007; Tao and Liu, 2016). However, L1 was generally used in participants’ daily lives and in their communication with the experimenters in these previous studies. In contrast, participants only used Dai to communicate within their own ethnic group in the present investigation, L2 (Han) was the more commonly used language. A previous survey from the Dai region showed that Dai students mostly use Han instead of Dai at school and in their daily lives (Yin, 2005). Thus, a possible explanation of such difference would be that Dai–Han NPBs are more proficient in Han than in Dai.

Green (1998) pointed out that the inhibitory effect is proportional to the activation level of words. In the present study, PB participants were proficient in both L1 and L2, which suggests that PBs may exercise more ability to inhibit the activation of a task-unrelated language, and to ensure the activation of task-related language successfully. The shorter RT of PBs indicates that their inhibitory control ability is greater. Notably, the result also shows that the effect of stimulus consistency is smaller for PBs than for NPBs. In other words, interference by Dai stimuli tends to cause differences in inhibitory control between PBs and NPBs at the attentional control stage, but not at the response control stage. This result is inconsistent with the second hypothesis. Bunge et al. (2002) claimed that an attentional bias caused by a conflict on stimulus level often occurs at the attentional control stage. The present study shows that the difference of inhibitory control ability is mainly influenced by proficiency of Dai, but not by the nonverbal response to the type or the position of the pictures. In this case, PBs seem to need more cognitive ability than NPBs to inhibit the interference of Dai words. Therefore, the difference of inhibitory control between PBs and NPBs occurs at the stage of attentional control under the Dai condition. Furthermore, as observed from the Inhibition Control Model (Green, 1998), the lexicon task schemes of both Dai and Han may have been activated, producing competition. The participants then have to follow the task-related scheme and inhibit the unrelated task scheme, that is, they have to inhibit the non-task-related information (i.e., the picture position and interference by Dai lexicons) to classify the picture. At the response inhibition stage, the participants have to inhibit the interfering picture location in their response to the stimuli. Bialystok (2007) pointed out that inhibitory control ability is mainly determined by the degree of cognitive resources employed in the processing. In the present study, the proficiency level of languages seems to be the main influence on lexicon selection. Thus, a weaker effect of response consistency indicates less interference by the picture location in the process of key-pressing, that is, less cognitive resources are utilized by both PBs and NPBs as compared with the interference of Dai stimuli. As a result there appears no difference between PBs and NPBs in their inhibition control ability at the response inhibition stage in Dai condition.

In Han condition, the effect of response consistency is smaller for PBs than for NPBs. In other words, Han stimuli interference could lead to differences at the response inhibition stage, but not at the attentional control stage. This pattern is opposite to Dai language (L1) and is also inconsistent with the third hypothesis. The results from Liu’s (2011) study indicated that the inhibitory control occurs at the attentional control stage under Han condition (L2 for Meng-Han bilinguals), which indicated that the proficiency of Han is higher in Han monolinguals than proficient Meng-Han bilinguals. In the present study, however, the inhibitory control occurs at the inhibitory control stage under L2 condition. According to Yin (2005), an explanation for such result would be that the PBs’ proficient level of Han language is as high as that of NPBs’. Probably, the difference of inhibitory control ability was not caused by the participants’ proficiency of Han since there was no difference of inhibitory control between PBs and NPBs at the attentional control stage. Bialystok and Majumder (1998) argue that the cognitive control abilities of PBs are greater than that of NPBs and assume that the proficiency of bilingual facilitates the development of cognitive control abilities. Liu (2011) also points out that, compared with monolinguals, bilinguals tend to have greater cognitive control abilities, which include verbal inhibitory ability (e.g., lexicon acquisition, abstract thinking, and language understanding) and nonverbal inhibitory ability (e.g., spatial discrimination). It seems that the advantage of cognitive control abilities is not only reflected in verbal inhibitory ability, but also includes nonverbal inhibitory ability. In this study, participants completed a spatial–dimensional task to determine whether or not the position of picture was in consistence with the position of response key. Specifically, the participants were asked to inhibit the interference of picture’s position to press the correct key to categorize the picture showed to them. In this case, the difference of inhibitory control between PBs and NPBs occurs at the response inhibitory stage because PBs have greater cognitive control abilities, especially the nonverbal inhibitory ability to inhibit the interference of picture’s position and thus pressing the corresponding key faster than NPBs.

Its also noteworthy that the present findings indicate that more cognitive resources are employed by Dai lexicon interference compared with Han lexicon interference as the participants were more proficient in Han than in Dai. It is possible that Dai and Han are subject to unbalanced development in the present bilingual samples. To verify this speculation, further investigations are needed focusing on linguistic developmental characteristics of Dai–Han bilinguals. Specifically, for most Dai children, Dai is their L1 and they have been living in a Dai-language-dominant environment from their birth to primary school, where they start a formal bilingual program to learn Han as L2. The primary schools in the Dai area on which the present study is based have implemented a bilingual teaching model called *Mastering Both the Chinese and Minority Languages* as promoted by the Yunnan Provincial Board of Education. As a result, Dai–Han bilingual students have been exposed to *the transitional bilingual teaching program*. During their first 2 years at school, Dai is the dominant instructional and communicative language. As they move on to higher levels (the third or the fourth grade, for instance), Han becomes the dominant language in their studies, with only a few courses taught in Dai. Currently, Han (L2) has been almost predominantly used as a communicative language both at school and in daily lives, and the chances for using Dai (L1) may grow rare. It can be expected that the participants to be selected from the third and fourth grades will probably be using Han exclusively. As a result, their L2 proficiency level will surpass their L1.

## Conclusion

The fact that PBs possess stronger inhibitory control ability than NPBs seems to suggest that bilingual acquisition can promote inhibitory control ability. In addition, there still exist differences of inhibitory control mechanism between L1 and L2 in lexicon selection during speech comprehension. In terms of higher language proficiency (usually for L1, but L2 in this study), the difference of inhibitory control between PBs and NPBs occurs in the stage of response inhibition. For the less proficient language [L2 generally, but Dai (L1) in this study], the difference of inhibitory control between PBs and NPBs occurs in the stage of attentional control. But the present study is limited in its selection of sample participants from Grades 3 and 4. Future research is needed to include a wider range of participants, covering Grades 1–6 so as to better clarify students’ language development in Dai–Han bilingual program, especially regarding at which time L1 advantage gives way to L2 advantage for Dai–Han bilinguals.

## Author Contributions

YT substantial contributions to the conception; revising it critically for important intellectual content; final approval of the version; agreement to be accountable for all aspects of the work. ZL design of the work and the analysis and interpretation of data for the work; revising it critically for important intellectual content; final approval of the version; agreement to be accountable for all aspects of the work. TT modify the design of the work; revising it critically for important intellectual content; final approval of the version; agreement to be accountable for all aspects of the work. RC design of the work; drafting the work; final approval of the version; agreement to be accountable for all aspects of the work. XM the acquisition of data; revising it critically for important intellectual content; final approval of the version; agreement to be accountable for all aspects of the work. XW the acquisition of data; revising it critically for important intellectual content. YL Polish the manuscript; revising it critically for important intellectual content. YQ the acquisition of data; revising it critically for important intellectual content; final approval of the version to be published; agreement to be accountable for all aspects of the work.

## Conflict of Interest Statement

The authors declare that the research was conducted in the absence of any commercial or financial relationships that could be construed as a potential conflict of interest.
