# Corrigendum: Inhibitory Control in Speech Comprehension among Dai–Han Bilingual Children

**DOI:** 10.3389/fpsyg.2017.01817

**Published:** 2017-10-19

**Authors:** Yun Tao, Zhi Liu, Tobias Tempel, Rui Chen, Xie Ma, Xiaoxi Wang, Yan Liu, Yongxia Qu

**Affiliations:** ^1^School of Educational Science and Management, Yunnan Normal University, Kunming, China; ^2^Key Laboratory of Educational Informatization for Nationalities, Ministry of Education, Yunnan Normal University, Kunming, China; ^3^Fachbereich I – Psychologie, University of Trier, Trier, Germany; ^4^College of Preschool Education and Special Education, Kunming University, Kunming, China; ^5^Faculty of Foreign Languages and Cultures, Kunming University of Science and Technology, Kunming, China

**Keywords:** Dai–Han bilinguals, proficient, primary school students, response inhibition, attentional control

There is an error in the Funding statement. The correct number for the National Natural Science Foundation of China is 31660282.

In the published article, the following affiliation should be added for Xiaoxi Wang: “College of Preschool Education and Special Education, Kunming University, Kunming, China.”

Also, the correct affiliation for Yan Liu should be: “Faculty of Foreign Languages and Cultures, Kunming University of Science and Technology, Kunming, China.”

The original article has been updated.

The authors apologize for these errors and state that this does not change the scientific conclusions of the article in any way.

## Conflict of interest statement

The authors declare that the research was conducted in the absence of any commercial or financial relationships that could be construed as a potential conflict of interest.

